# Antioxidants and Oxidative Stress: Focus in Obese Pregnancies

**DOI:** 10.3389/fphys.2018.01569

**Published:** 2018-11-06

**Authors:** Martin Alcala, Sebastián Gutierrez-Vega, Erica Castro, Enrique Guzman-Gutiérrez, Maria Pilar Ramos-Álvarez, Marta Viana

**Affiliations:** ^1^Department of Chemistry and Biochemistry, Facultad de Farmacia, CEU San Pablo University – CEU Universities, Madrid, Spain; ^2^Red Iberoamericana de Alteraciones Vasculares Asociadas a TRastornos del EMbarazo (RIVA-TREM), Chillán, Chile; ^3^Molecular Medicine Laboratory, School of Medical Technology, Faculty of Health Sciences, Universidad San Sebastián, Concepción, Chile; ^4^Faculty of Medicine, Universidad San Sebastián, Concepción, Chile

**Keywords:** fertility, preeclampsia, miscarriage, latin-America, teratogenesis, intrauterine growth restriction, oxidative stress

## Abstract

The prevalence of obesity in women of childbearing age around the globe has dramatically increased in the last decades. Obesity is characterized by a low-state chronic inflammation, metabolism impairment and oxidative stress, among other pathological changes. Getting pregnant in this situation involves that gestation will occur in an unhealthy environment, that can potentially jeopardize both maternal and fetal health. In this review, we analyze the role of maternal obesity-induced oxidative stress as a risk factor to develop adverse outcomes during gestation, including reduced fertility, spontaneous abortion, teratogenesis, preeclampsia, and intrauterine growth restriction. Evidences of macromolecule oxidation increase in reactive oxygen species generation and antioxidant defense alterations are commonly described in maternal and fetal tissues. Thus, antioxidant supplementation become an interesting prophylactic and therapeutic tool, that yields positive results in cellular, and animal models. However, the results from most meta-analysis studying the effect of these therapies in complicated gestations in humans are not really encouraging. It is still to be analyzed whether these therapies could work if applied to cohorts of patients at a high risk, such as those with low concentration of antioxidants or obese pregnant women.

## Prevalence of Overweight and Obesity Among Women of Reproductive Age

Prevalence of obesity in both developed and developing countries has increased among women over the last decades (Heslehurst et al., 2007; NCD Risk Factor Collaboration, 2016), including the prevalence in women of childbearing age, which has also raised dramatically worldwide (Nguyen et al., 2008; Menting et al., 2018; Schaefer-Graf et al., 2018), from 29.8% in 1980 to 38.0% in 2013 (Ng et al., 2014). Correa and Marcinkevage reported an average global prevalence of obesity in women of childbearing age ranging between 1% in Chad to 70.3% in Tonga (Correa and Marcinkevage, 2013).

**FIGURE 1:**
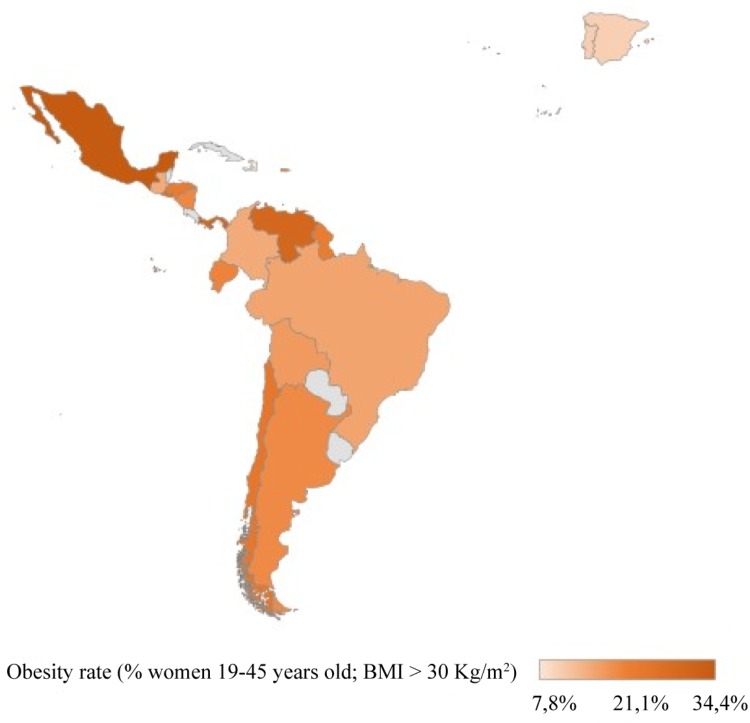
Estimates of obesity in women of childbearing age in Latin-America. Percentage of obesity (BMI ≥ 30 Kg/m^2^) in women between 18 and 45 years old. BOL, BRA, COL, ECU, ESV, GUA, HAI, HON, MEX, NIC, PER, and GUY obesity prevalence obtained from National Health Surveys and compilated in (Correa and Marcinkevage, 2013). VEN prevalence obtained from Instituto Nacional de Nutrición (2008–2010). CHI obesity prevalence obtained from Encuesta Nacional de Salud (ENS-2009) and reviewed in (Araya et al., 2014). PAN obesity prevalence obtained from cross-sectional studies (2010–2011) (Mc Donald et al., 2015). ARG obesity prevalence obtained from Encuesta Nacional de Nutrición y Salud (ENNyS-2007). PR obesity prevalence obtained from self-reported, prospective longitudinal cohort study (2011–2014) (Guilloty et al., 2015). SPA and POR obesity prevalence obtained from the European Health Interview Survey (EHIS 2014).

The available epidemiological data in Latin-American countries are dispersed, obtained from periodic national surveys in some cases, or from low-scale, punctual regional studies in others. As it is shown in the Figure 1, the highest prevalence of obesity in women of reproductive age was found in Mexico, while the lowest prevalence was found in Haiti.

The trend in the prevalence of obesity has become a matter of concern to preconception healthcare programs because pre-pregnancy obesity and excessive GWG is associated with an increased risk of adverse reproductive health outcomes (Dolin and Kominiarek, 2018; Most et al., 2018). Obesity reduces fertility and increases time taken to conceive (Poston et al., 2016). At the beginning of the gestation, obese pregnant women are more likely to have spontaneous and recurrent pregnancy loss (Chu et al., 2007). The rate of embryo malformations is also increased, showing mainly neural tube, and cardiac defects (Rasmussen et al., 2008). During mid-late gestation, obesity pregnancies have an increased risk for PE and gestational diabetes mellitus, both of which are associated with long-term morbidities post-partum (Milne et al., 2009; Poston et al., 2016). Obese women can also experience difficulties during labor and delivery. The chances of requiring a cesarean intervention in obese mothers double that of lean mothers, while the comorbidity (anesthetic complications, massive blood loss) reaches almost 34% of the gestations. For the newborn, there is a higher risk of LGA, macrosomia, shoulder dystocia and even obesity in childhood (Dennedy and Dunne, 2010; Nelson et al., 2010; Most et al., 2018).

Obesity is associated with a dysregulation in the metabolic balance comprising lipid metabolism, inflammatory or hormonal processes in addition to insulin resistance (Bozkurt et al., 2016). The pathogenesis of obesity is complex and includes metabolic and hormonal dysregulation, low-grade chronic inflammation and endoplasmic reticulum stress, among other processes that are closely interconnected. Several groups all over the world, including ourselves (Alcala et al., 2015), have focused our research in the role of oxidative stress as a central mechanism that may enhance the aforementioned conditions. In this context, we have shown how the use of antioxidants in long-term obesity reduces obesity-associated inflammation, insulin resistance and tissue fibrosis. In the following lines we will discuss the role of OS before, during and after gestation in the mother with pregestational obesity and in the offspring, reviewing the use of antioxidant therapies to ameliorate or prevent undesired negative outcomes.

## Oxidative Stress

OS has been traditionally recognized as a key factor in the pathophysiology of numerous conditions, including cardiovascular and neurodegenerative diseases, cancer, diabetes, and obesity.

OS has been classically defined as an imbalance between ROS generation and its detoxification by antioxidant systems, in favor of the former. ROS are partially reduced, oxygen-containing metabolites (some of them are free radicals) that are generated because of normal cellular metabolism and environmental factors. They are extremely reactive and have the potential to oxidize lipids, proteins and DNA. On the other side, enzymatic (superoxide dismutase, catalase, glutathione peroxidase) and non-enzymatic (vitamin C and E, glutathione) antioxidants neutralize the effect of highly reactive ROS by transforming them into less reactive species and eliminating oxidation by-products, protecting cells from oxidative damage.

However, ROS, at a physiological concentration, behave as second messengers in several signaling pathways that are critical for the normal cellular function. Several studies in the last 10 years have described how ROS can modify the redox state of key residues of proteins (Jones et al., 2004) regulating enzyme activity. They have been reported to participate in different signaling pathways (NF-κB, PTP1b, ASK1, PTEN, REF1, p66hc, or IRP1) (Ray et al., 2012).

Adipose tissue has been proposed as the origin of obesity-induced OS, that is later transmitted to other tissues and may account for obesity-associated diseases such as hypertension, cardiovascular disease, or even cancer (Furukawa et al., 2004; Matsuda and Shimomura, 2013). Hyperglycemia, hyperleptinemia, endothelial dysfunction, hyperlipidemia and mitochondrial dysfunction are the main mechanisms that have been described to increase ROS-generation systems such as nitric oxide synthases (NOS) or NADPH oxidases (Savini et al., 2013). In the first stages of obesity, an upregulation of the antioxidant enzymes is observed to prevent oxidative damage, but as fat accumulates the antioxidant defense is overtaken, leading to OS (Alcala et al., 2015; Alcala et al., 2017).

In addition, oxidative stress has also been observed in healthy gestation. In the second trimester, there is a spike in oxygen supply and metabolic rate in placenta. If ROS levels are maintained under control, they regulate trophoblast proliferation, invasion and angiogenesis, required for a healthy pregnancy (Wu et al., 2015).

Thus, both obesity and gestation are characterized by an increased OS. When combined, OS is one of the proposed mechanisms involved in many reproductive and pregnancy disorders that may lead to adverse pregnancy outcomes (Malti et al., 2014).

## Oxidative Stress, Maternal Obesity and Pregnancy Outcomes

### Fertility

Pre-existing obesity is an independent risk factor for anovulation, subfertility and infertility in women (Silvestris et al., 2018). Several studies show a positive correlation between maternal BMI and time-to-pregnancy (Gesink Law et al., 2007; Wise et al., 2010). It is estimated that for every unit of increase in the BMI, there is a 5% decrease in the probability of conception (van der Steeg et al., 2008). On the other hand, weight loss strategies may positively influence in fertility (Sim et al., 2014). A 10% of weight loss in overweight patients with infertility significantly improved conception and live birth rates (Kort et al., 2014).

The deleterious effect of obesity on reproduction is mainly driven by endocrine and metabolic alterations, which may interfere with the neuroendocrine and ovarian function through a disruption in the hypothalamus-hypophysis-ovarian axis. The mechanisms underlying the defective endocrine program in obese women include metabolic alterations due to hyperinsulinemia, the effect of pro-inflammatory cytokines, endoplasmic reticulum stress, alterations in the mitochondria and OS (Silvestris et al., 2018).

In rodents, oocytes from dams fed on high fat diet showed abnormal mitochondrial morphology and increased activity, resulting in increased ROS production and GSH reduction (Igosheva et al., 2010) leading to meiosis failure (Han et al., 2017; Wang et al., 2018). Circulating markers of OS are also elevated in women with polycystic ovary syndrome, with a remarkable 50% decline in GSH concentration. In fact, GSH seems to be a critical player in both male and female fertility. The lack of the enzyme that catalyzes the rate-limiting step in GSH synthesis, the glutamate cysteine ligase, in female mice dramatically reduced preimplantation development (Nakamura et al., 2011; Lim et al., 2015).

The use of antioxidants to improve fertility in obese patients is still a matter of debate. A recent meta-analysis concludes that there is low-quality evidence of a beneficial effect of antioxidants to increase fertility (Showell et al., 2017). However, this report fails to independently analyze a cohort of women with pre-gestational obesity, where the oxidative insult may arise from the combination of two situations that are, independently, associated to oxidative stress. In this situation, the use of antioxidants such as resveratrol in preclinical studies using rodent models (Ghowsi et al., 2018; Jia et al., 2018), or in randomized control trials in humans using Mg and Zn (Afshar Ebrahimi et al., 2017) or NAC (Nasr, 2010; Maged et al., 2015) improved overall reproductive outcome.

### Miscarriage and Obesity

Overweight and obese pregnant women present a higher risk of pregnancy loss and recurrent miscarriage compared to normal weight gestations (OR vary from 1.31 to 1.67) (Metwally et al., 2008; Boots and Stephenson, 2011). There is an even higher risk for obese women of recurrent early miscarriage in spontaneous conception (OR: R: 3.51; 95% CI, 1.03–12.01) and miscarriage after ovulation induction (OR: 5.11; 95% CI, 1.76–14.83).

Recent results suggest a reduced regenerative capacity and plasticity of the endometrium in obese pregnant women (Murakami et al., 2013), factors that may predispose for pregnancy loss (Lucas et al., 2016).

Another key step for a successful pregnancy is a proper maternal-fetal exchange. From week 8 to 12 of gestation there is a peak in placental pO_2_ when the maternal blood enters the placenta. This signal triggers the transcription of antioxidant genes (catalase, glutathione peroxidase, and superoxide dismutase) to overcome the prooxidant environment (Jauniaux et al., 2000). In obese pregnancies, together with a pre-established oxidative situation (Catalano and Shankar, 2017), there is a dysregulation of immune cells within the endometrium, characterized by a reduced presence of the anti-inflammatory Treg lymphocytes and an increase of natural killer lymphocytes (Quenby et al., 2009). This promotes early angiogenesis, increasing placental pO_2_ prior to the establishment of a mature antioxidant system, depleting non-enzymatic antioxidants such as GSH and vitamin E (Hansen and Harris, 2013). Several authors have described an increase in OS markers in placenta from early and recurring pregnancy loss and suggested that increased ROS generation may be caused by premature establishment of maternal placental perfusion, which has been correlated with a higher risk of miscarriage (Miller et al., 2000; Burton and Jauniaux, 2004; Yiyenoglu et al., 2014).

However, current meta-analyses have shown no beneficial effect of the administration of vitamins (alone or in combination) prior to pregnancy or during the early stages of pregnancy (Balogun et al., 2016). Given the importance of GSH metabolism to fully develop a successful pregnancy, more studies should be carried out to evaluate the potential of NAC (a GSH precursor) to prevent obesity-related miscarriage. So far, the supplementation with NAC to women with recurrent unexplained pregnancy loss significantly increased the rate of living pregnancies beyond 20 weeks and the take-home baby rate (Amin et al., 2008).

### Malformation

Congenital anomalies are the end-products of aberrant organogenesis in utero during the first trimester of gestation. A meta-analysis in 2009 revealed that newborns from obese mothers are at increased risk of severe congenital malformations, including neural tube defects and cardiovascular anomalies (Stothard et al., 2009). Results from a cohort including more than 1.2 million deliveries, showed that liveborn singletons from mothers with a BMI > 40 kg/m^2^ almost double the risk of suffering major congenital malformations in the nervous system compared to the offspring of normal weight mothers (Persson et al., 2017).

The mechanisms involved in obesity-mediated teratogenesis are still unrevealed. Traditionally, the fuel-mediated teratogenesis hypothesis claims that exposing the embryo to an excessive amount of nutrients, mainly glucose and ketone bodies, may promote embryo malformations, inappropriate organ development and metabolic disturbances in the youth (Freinkel, 1980; Catalano, 2010; Plagemann and Harder, 2011).

OS, common feature in maternal obesity (Gallardo et al., 2015), has been suggested as a potential mechanism in the teratogenesis induced by diabetes (Viana et al., 1996) or chemical teratogens such alcohol, cocaine, valproate, or thalidomide (Hansen and Harris, 2013).

In addition to a direct effect on DNA damaging and repair (Wells et al., 2010), at a molecular level, OS has been shown to inhibit *Pax3* upregulation during early embryogenesis in a murine embryonic stem cell model (Wu et al., 2012). *Pax3* is a transcription factor required for neural tube development. As a result, cardiac neural crest and neuroepithelial cells undergo apoptosis by a process dependent on the p53 tumor suppressor protein (Wang et al., 2011). The supplementation with GSH and vitamin E has proven to be effective in the upregulation of Pax3 expression after an oxidative insult (Wu et al., 2012).

To the best of our knowledge, there is not any clinical trial, to test the potential protective effects of antioxidant supplementation in pre-gestational obese women. However, using retrospective, survey-based studies, a reduction in antioxidant consumption has been linked to increase odds of limb and neural tube defects in obese pregnant women (Chandler et al., 2012; Pace et al., 2018).

### Preeclampsia and Cardiovascular Alterations

PE is a severe disease characterized by the presence of hypertension and proteinuria during the second and third trimester of gestation (Steegers et al., 2010). PE affects approximately 2–8% of all pregnancies (Ghosh et al., 2014) and is associated with substantially higher maternal and fetal morbidity and mortality worldwide, especially in Latin American countries where PE is one of the leading causes of maternal and fetal mortality (Giachini et al., 2017).

PE women exhibit at least a twofold increased risk of stroke, while risk of death due to ischemic heart disease is eight times higher when PE occurs before 34 weeks of gestation (Deanfield et al., 2005). Indeed, the American Heart Association has included PE as a risk factor for future cardiovascular disease (Bushnell et al., 2014).

Obesity has been listed as a major risk factor for PE (Marchi et al., 2015), together with higher waist circumference, blood pressure, insulin, proinsulin, glucose, C-reactive protein and triglycerides levels, and lower HDL cholesterol (Mongraw-Chaffin et al., 2010; Sliwa and Bohm, 2014).

The current well-accepted pathophysiology of PE involves a two-stage model: first, an incomplete remodeling of the spiral arteries communicating maternal and placental blood flow through a defective trophoblast invasion. This leads to the second stage, where the ischemia-reperfusion cycles triggers the release of harmful molecules including ROS, cytokines, antiangiogenic proteins, cell fragments, microparticles, and extracellular vesicles (Redman and Sargent, 2000, 2005; Tannetta et al., 2013; Hod et al., 2015). These elements may reach the maternal circulation and are thought to be the causative factors of endothelial dysfunction (Roberts and Escudero, 2012).

OS, common in obesity, appears to be the central component of both placental and endothelial dysfunction (Aouache et al., 2018). Pregestational obesity modifies the arterial architecture in placenta (Avagliano et al., 2012) and its contraction/relaxation capacity, being more susceptible to maternal OS (Saker et al., 2008). In addition, obese placenta presents an increased generation of mitochondrial ROS caused by a defective respiratory chain (Hastie and Lappas, 2014), that has been linked to placental angiodysplasia (Ishii et al., 2014). The following episodes of hypoxia/reoxygenation induce the activity of the xanthine oxidase, an important source of superoxide (Hung et al., 2002).

Oxidative damage in the placenta leads to inflammation, apoptosis and the release of cellular debris into maternal circulation, along with several anti-angiogenic factors, cytokines and oxidants (MDA, isoprostanes) concomitant with a reduced antioxidant capacity (Atamer et al., 2005). These placental-derived factors act on the maternal vascular endothelium, inducing more OS and stimulating the production and secretion of pro-inflammatory cytokines, as well as vasoactive compounds. This results in a massive systemic endothelial dysfunction characterized by vascular inflammation and constriction (Goulopoulou and Davidge, 2015).

Preclinical experiments in cellular and animal models reported beneficial effects of antioxidants reducing maternal blood pressure and improving endothelial function (Chang et al., 2005; Roes et al., 2006; McCarthy and Kenny, 2016). However, meta-analysis from clinical trials in humans did not support the use of antioxidant therapy to reduce the risk of PE (Rumbold et al., 2008, 2015; Roberts et al., 2010). It is important to notice that none of these reviews stratify the population according to the BMI, so the effect of antioxidants on PE women with a preexisting oxidative situation has not been studied yet.

### Intrauterine Growth Restriction and Obesity

An adequate transport of O_2_ and nutrients in the mother-placenta-fetus circuit is mandatory for the normal development of gestation. An excess in the nutrient supply from obese mothers (Rosario et al., 2015) has been strongly associated with alterations in fetal growth (Yu et al., 2013), increasing the risk of delivering LGA (Bove et al., 2014). Strikingly, epidemiological studies have also noticed that SGA deliveries are also more frequent among overweight and obese women (Radulescu et al., 2013). It is still to be confirmed whether pregestational BMI or GWG have more impact on fetal growth (Crane et al., 2009). In any case, both pregnancy outcomes, SGA and LGA, have an increased risk of suffering perinatal complications, including stillbirth (Yao et al., 2017) and complications later in life.

LGA newborns are the result of an increased flux of oxygen and nutrients together with placental overgrowth (Gaccioli et al., 2013), with a feature gene expression and more OS than those AGA (Saker et al., 2008). Even several years after delivery, in a prepubertal age, LGA children present more OS and insulin resistance than AGA (Chiavaroli et al., 2009).

On the other hand, IUGR in obesity is partly caused by a defective oxygen and nutrient supply to the placenta, which resembles the pathological basis of PE (Srinivas et al., 2009). However, not every case of IUGR can be explained by preexisting PE, so the presence of divergent molecular mechanisms has been proposed by some authors (Villar et al., 2006).

Nonetheless, OS is present in both conditions and may have a critical impact on the development of the disease. In fact, an increase in oxidative markers (MDA, isoprostanes, protein carbonyls) has been found in placenta, maternal and chord plasma (Longini et al., 2005; Biri et al., 2007; Zadrozna et al., 2009; Mert et al., 2012; Negro et al., 2017) of IUGR-complicated pregnancies, with and without PE. Enzymatic antioxidant defenses are also upregulated, as an increase in superoxide dismutase or glutathione peroxidase activities have been previously described. However, non-enzymatic antioxidants, such as GSH, vitamin E and C contents are depleted (Biri et al., 2007; Rajasingam et al., 2009; Zadrozna et al., 2009; Mert et al., 2012), reflecting an OS situation.

To our knowledge, there is not any published clinical trial in humans to determine the potential effect of antioxidants (vitamins or GSH-precursors) on the intrauterine growth from obese mothers. Retrospective, food intake survey-based studies, showed conflicting results. For example, in Spain no relation was found between the consumption of antioxidant vitamins and SGA frequency (Salcedo-Bellido et al., 2017) and a meta-analysis about the use of vitamin E during pregnancy showed no beneficial effect to prevent poor fetal growth in healthy pregnancies (Cohen et al., 2015; Rumbold et al., 2015).

## Conclusion

**FIGURE 2:**
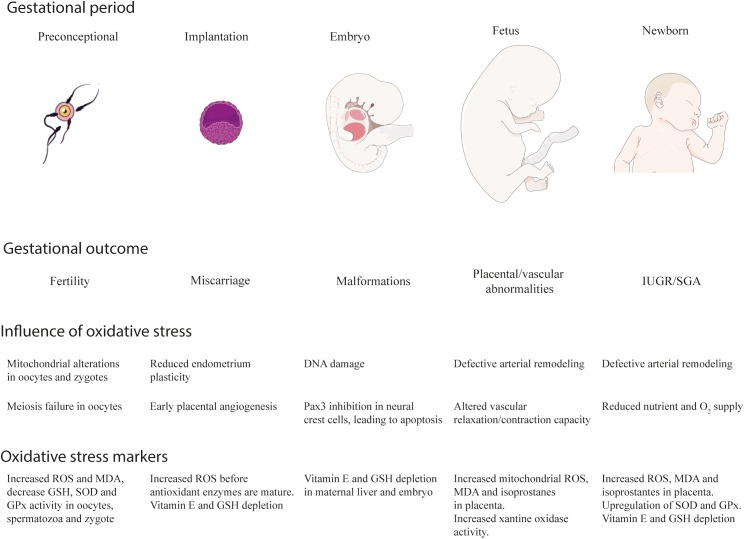
Obesity, gestation and oxidative stress. Pregestational obesity increases the risk of suffering adverse outcomes prior (reduced fertility), during (spontaneous abortion, embryo malformations, preeclampsia, intrauterine growth retardation) and after gestation (small and large for gestational age deliveries) for both the mother and the fetus. Oxidative stress is a common feature in the etiology of obesity and can cause an accumulative effect with the oxidative stress that appears during gestation. The effects and the evidences of oxidative stress in obesity-complicated pregnancies are here summarized. IUGR, intrauterine growth retardation; SGA, small for gestational age; ROS, reactive oxygen species; MDA, malondialdehyde. Artwork was obtained from Servier Medical Art, licensed under a Creative Common Attribution 3.0 Generic License. http://smart.servier.com/.

Pregestational obesity affects approximately to 1 out of 3 women of childbearing age in the world. The excessive fat mass accumulation correlates with OS, caused by an overactivation of the ROS-generating systems (mainly NOS and NADPH oxidases) and a depleted antioxidant defense. Obesity-induced OS arises as a central factor that increases the risk of adverse outcomes in gestation, as it has been summarized in the Figure 2. Prior to gestation, obesity-related OS can cause decreased fertility due to a defective meiosis and mitochondrial alterations in the oocytes. On early gestation, OS increases the risk of miscarriage by reducing the plasticity of the endometrium and promoting early angiogenesis, that increases oxygen supply prior to the maturation of the antioxidant systems. OS can directly cause DNA damage and the inhibition of key genes for neural tube development and closure, responsible for some of the most common malformations observed in embryos from obese mothers. During the second trimester, placenta becomes another physiological source of ROS, with a physiological role on materno-fetal connection. However, the combination of both sources of OS in obese pregnancies can cause an overproduction of ROS, that may account for a defective vascularization of the placenta, leading to both hyperoxia and hypoxia. These two situations exacerbate the placental oxidative state and participate in the pathology of vascular conditions such as PE and IUGR.

Despite the multiple evidence of the oxidative disbalance along normal pregnancies, the use of antioxidants to prevent these outcomes is conflicting. While they have proven to be effective in preclinical studies in cellular and animal models, the reports of its application in large-scale clinical trials is often discouraging. However, the analyzed clinical trials in this mini-review do not specifically analyze a cohort of women with pregestational obesity. The design of specific clinical trials for this population, with a basal situation of increased OS, could potentially generate more promising results. Besides clinical recommendations to obese women, such as weight loss before conception and controlling GWG, specific studies on antioxidant therapies focused on this population could help clarifying the adequacy of targeting OS to prevent complications.

## Author Contributions

MA and MV participated in the conception of the work and in the preparation of the manuscript. SG-V, EC, EG-G, and MR-Á participated in the preparation of the manuscript and critically reviewed the final draft.

## Conflict of Interest Statement

The authors declare that the research was conducted in the absence of any commercial or financial relationships that could be construed as a potential conflict of interest.

## Abbreviations

AGA: appropriate for gestational age
GSH: glutathione
GWG: gestational weight gain
IUGR: intrauterine growth restriction
LGA: large for gestational age
MDA: malondialdehyde
NAC: N-acetylcysteine
OR: odds ratio
OS: oxidative stress
PE: preeclampsia
ROS: reactive oxygen species
SGA: small for gestational age
